# Body ownership and agency altered by an electromyographically controlled robotic arm

**DOI:** 10.1098/rsos.172170

**Published:** 2018-05-09

**Authors:** Yuki Sato, Toshihiro Kawase, Kouji Takano, Charles Spence, Kenji Kansaku

**Affiliations:** 1 Systems Neuroscience Section, Department of Rehabilitation for Brain Functions, Research Institute of National Rehabilitation Center for Persons with Disabilities, 4-1 Namiki, Tokorozawa, Saitama 359-8555, Japan; 2 Research Organization of Science and Technology, Ritsumeikan University, 1-1-1 Noji-higashi, Kusatsu, Shiga 525-8577, Japan; 3 Institute of Innovative Research, Tokyo Institute of Technology, 4259 Nagatsuta, Midori, Yokohama, Kanagawa 226-8503, Japan; 4 Institute of Biomaterials and Bioengineering, Tokyo Medical and Dental University, 2-3-10 Kanda-Surugadai, Chiyoda, Tokyo 101-0062, Japan; 5 Crossmodal Research Laboratory, Department of Experimental Psychology, Oxford University, Oxford OX1 3UD, UK; 6 Brain Science Inspired Life Support Research Center, The University of Electro-Communications, 1-5-1 Chofugaoka, Chofu, Tokyo 182-8585, Japan; 7 Department of Physiology and Biological Information, Dokkyo Medical University School of Medicine, 880 Kitakobayashi, Mibu, Tochigi 321-0293, Japan

**Keywords:** rubber hand illusion, ownership, agency, body representation, robotic arm

## Abstract

Understanding how we consciously experience our bodies is a fundamental issue in cognitive neuroscience. Two fundamental components of this are the sense of body ownership (the experience of the body as one's own) and the sense of agency (the feeling of control over one's bodily actions). These constructs have been used to investigate the incorporation of prostheses. To date, however, no evidence has been provided showing whether representations of ownership and agency in amputees are altered when operating a robotic prosthesis. Here we investigated a robotic arm using myoelectric control, for which the user varied the joint position continuously, in a rubber hand illusion task. Fifteen able-bodied participants and three trans-radial amputees were instructed to contract their wrist flexors/extensors alternately, and to watch the robotic arm move. The sense of ownership in both groups was extended to the robotic arm when the wrists of the real and robotic arm were flexed/extended synchronously, with the effect being smaller when they moved in opposite directions. Both groups also experienced a sense of agency over the robotic arm. These results suggest that these experimental settings induced successful incorporation of the prosthesis, at least for the amputees who took part in the present study.

## 1. Introduction

A fundamental issue in cognitive neuroscience involves trying to understand how it is that we consciously experience our bodies. The experience of the body as one's own is termed the ‘sense of body ownership’ (SO), and the conscious experience that one is initiating and controlling one's own volitional actions is termed the ‘sense of agency’ (SA). These two aspects of body consciousness have been investigated in neuroscience, psychology and philosophy for many decades now because they constitute core components of the conscious experience of self [1–3].

In certain circumstances, the SO is extended outside of our own body, as in the well-known rubber hand illusion (RHI) [4–10]. In RHI experiments, both a visible rubber hand and the participant's own hidden hand are typically stroked in synchrony with paintbrushes. Watching the rubber hand can elicit the feeling that the rubber hand is one's own. However, *asynchronous* stimulation of the two hands typically reduces or eliminates the illusion. Thus, the congruency between visual and tactile information is thought to be important in eliciting the sense of ownership.

Recent RHI research using moving rubber hands indicates that just as the SO can be extended outside of our own body, so too can the SA [11–18]. For example, in one study, the index fingers of the participant's hand and the rubber hand were connected by a rod so that the participants could control the movements of the index finger of the rubber hand by moving their own index finger. The results of this study once again revealed that the participants experienced the SO and SA over the rubber hand in the synchronous condition [13].

Upper limb amputees can also experience a rubber hand as part of their own body upon the application of synchronous touches to their stump and the artificial hand [19,20]. For instance, Marasco *et al*. [21] demonstrated that amputees experienced a SO over a prosthetic limb by applying physiologically appropriate cutaneous feedback from the prosthetic limb. In addition, Rosen *et al*. demonstrated that amputees experienced a SO over an artificial hand by applying synchronous touches to the participant's stump and the artificial hand. They also demonstrated that the amputees experienced a SO over the artificial hand when it was controlled by the activity of their own arm muscle [22].

For amputees, it is desirable that a prosthesis be incorporated successfully into their body representation [23], so that they recognize it as part of their own body, and can move it in accordance with their own volition. In the present study, we developed a robotic arm using proportional myoelectric control, for which the user could vary the joint positions continuously, in an RHI task. Electromyographic signals were recorded from the arm of each participant, and were used to control the robotic arm, placed in front of them. Our experiments revealed significant increases in subjective ratings of the SO and SA, not only in the able-bodied participants but also in the amputees.

## 2. Material and methods

### 2.1. Able-bodied participants

#### 2.1.1. Participants

In total, 15 able-bodied participants (aged 33.53 ± 3.75 years, range 25–39; 12 females) were recruited. All of the participants were neurologically normal and right-handed [24].

#### 2.1.2. Experimental settings for the robotic arm

The robotic arm, using proportional myoelectric control [25] with one degree of freedom (wrist flexion and extension), consisted of a prosthetic glove (model 8S11N; Ottobock, Duderstadt, Germany) and an actuator (model FHA-11 C; Harmonic Drive LLC, Peabody, MA). The prosthetic glove was similar to a real hand in terms of its size, shape and appearance. The joint positions of the robotic arm were controlled continuously by means of the participant's muscular activity via two electrodes (model DE-2.1; DELSYS, Natick, MA) capturing electromyographic (EMG) signals from the wrist flexor (flexor carpi radialis muscle) and the wrist extensor (extensor carpi ulnaris muscle). Each signal was sampled at 2000 Hz. To extract information on muscular activation from the EMG signals, the signal was rectified digitally and low-pass filtered [26]. Calibration of the robotic arm was performed using a computer-based algorithm in Matlab (MathWorks Inc., Natick, MA) using a two-layer feed-forward neural network [27]. In the calibration phase, the participants alternately flexed and then extended their wrist a few times synchronously with the robotic hand movement. The computer-based algorithm identified parameters of the neural network for estimating the participant's wrist angle using these EMG signals. After calibration, the flexion/extension of the participant's wrist resulted in a simultaneous congruent movement of the robotic arm.

#### 2.1.3. Procedure

**Figure 1.**
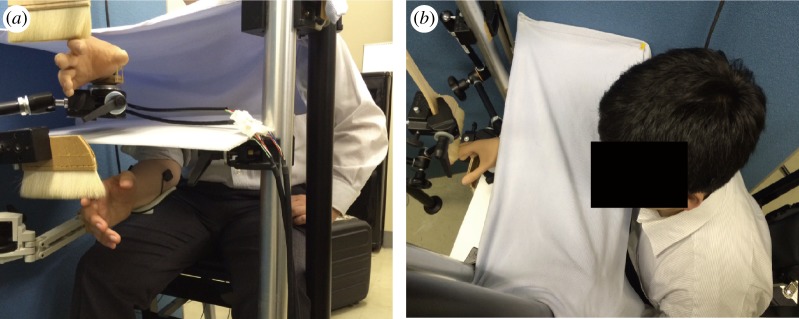
Experimental set-up for the able-bodied participants. (*a*) A plastic board was placed horizontally in front of the participant, and each participant placed their right arm under the board. EMG signals were recorded from each participant's arm to control the robotic arm, placed above the board. The robotic arm and the participant's hand were stroked with paintbrushes. The wrist of the robotic arm was flexed/extended when the participant's wrist flexors/extensors were contracted. (*b*) The robotic arm was positioned in front of the participant, while their right arm was kept hidden from their view.

Able-bodied participants sat in front of a plastic board (figure 1*a*). The robotic arm was placed above the board. The participant's right arm was placed directly 18 cm below the robotic arm, and was kept out of the participant's view throughout the course of the experiment. A cloth was placed over the participant's right arm to cover the space between the robotic arm and the participant (figure 1*b*). The participants took part in the in-phase and out-of-phase movement experimental conditions for 10 min each. They also participated in the experimental conditions (with and without the paintbrush). The paintbrush was added because a former study by Rosen *et al*. [22] found that additional tactile stimulation with the brush affected the illusion of ownership. The order in which these various conditions were conducted was counterbalanced across participants. In the conditions, each participant was instructed to flex/extend the wrist alternately, and to watch the robotic arm moving. In the in-phase movement condition, the wrists of the real and robotic arm were flexed and extended synchronously. In the out-of-phase movement condition, they moved in opposite directions. In the ‘with paintbrush’ condition, a paintbrush was placed above the robotic arm and tactile stimulation was delivered when the participants moved their hand. In the ‘without paintbrush’ condition, no paintbrush was used.

#### 2.1.4. Psychological evaluation

Measurements of embodiment were taken immediately after exposure to each experimental condition. The participants answered a questionnaire with three ownership statements, three agency statements and three control statements to assess both ownership and agency [15]. The participants rated their experience on a seven-point Likert scale ranging from −3 (totally disagree) to +3 (totally agree), with 0 indicating ‘uncertainty’. Average scores from each of the three ownership statements and from the three agency statements were computed. These average statement scores will be referred to as ‘ownership rating’ and ‘agency rating’. In addition, as in a previous study that tested for the elicitation of the RHI, we compared the category of ownership statements and the category of agency statements to the respective control category of statements for each condition separately.

We also tested the degree to which the participants felt that their right arm was located closer to the robotic arm after the illusion; this is known as proprioceptive drift, a response measure that is commonly used in RHI research. With their eyes closed, the participants pointed to indicate the sensed position of the right index finger with their left index finger. The participants had to make a pointing movement by touching a pole placed vertically in front of them on which the experimenter could mark the endpoint of each pointing movement. We measured the position pointed to before (pre-pointing) and after (post-pointing) each 10-min experiment. Proprioceptive drift in the vertical plane was then calculated by subtracting the two position measurements from each other (post- minus pre-pointing). Positive values indicated an upward drift of hand position, towards the robotic arm.

#### 2.1.5. Data analyses

Subjective ratings of the SO and SA were computed [15]. Our *a priori*-defined criterion for experiencing illusory ownership or agency in the given condition was that the median group score on the ownership or agency ratings was significantly greater than 0. We also compared the subjective ratings of body ownership seen in the in-phase movement condition with that seen in the corresponding out-of-phase movement condition. Similarly, the agency ratings in the phase and out-of-phase movement conditions were also compared.

Positive proprioceptive drift indicated an upward drift in the sensed hand position towards the robotic arm. Our *a priori*-defined criterion for upward drift was that the median group score would be significantly greater than 0.

For statistics, we used Wilcoxon signed-rank test at the 5% significance level.

### 2.2. Amputees

#### 2.2.1. Participants

Three trans-radial amputees (#1, #2, #3) took part in the study (table 1 for details). Participant #1 was a 67-year-old male who had had his right arm amputated because of trauma 15 years previously, and the stump length from the elbow was 20 cm. Participant #2 was a 40-year-old female, who had had her right arm amputated for sarcoma 5 years previously, with a 10 cm stump length from the elbow. Participant #3 was a 67-year-old male, who had had his left arm amputated because of trauma 61 years previously. In this case, the stump length from the elbow was 10 cm. In their daily lives, these amputees had not used myoelectric hands, but participant #1 used both a functional prosthetic arm and a cosmetic prosthetic arm, while participants #2 and #3 used a cosmetic prosthetic arm. Participant #1 experienced phantom sensation in the amputated limb. The phantom hand was felt as if it had been pushed completely inside the residual limb (telescoped phantom). Participant #1 could not move his phantom. Numbness was reported in the phantom thumb, but no pain was reported.

**Table 1.** Demographic and clinical characteristics of amputee participants.

participant (gender, age)	handedness	cause of amputation	time since amputation (years)	lower arm stump length (cm)	type of prosthesis	phantom limb	phantom pain
#1 (m, 67)	R	trauma	15	20	functional and cosmetic	yes	no
#2 (f, 40)	R	sarcoma	5	10	cosmetic	no	no
#3 (m, 67)	R	trauma	61	10	cosmetic	no	no

#### 2.2.2. Experimental setting for the robotic arm in amputees

We used the same robotic arm as for the able-bodied participants. EMG measurements were performed from the amputees' stumps. We mounted two electrodes: one for detecting residual muscle activities from the wrist flexor, the other for detecting residual muscle activities from the wrist extensor. The robotic arm was controlled with in-house software in Matlab using a linear transformation function from the EMG signals to estimate the desired angle of the robotic arm. A calibration of the linear transformation function was performed at the beginning of the experiment. Participants contracted the flexor/extensor muscles for wrist movement alternately at the maximum voluntary contraction. EMG signals were then normalized to the maximum EMG activity during the generation of maximum voluntary contraction. We manually identified parameters of the linear transformation function in order to estimate the desired wrist angle using the EMG signals. After the calibration phase, the contraction of the flexor/extensor muscles resulted in a simultaneous congruent movement of the robotic arm.

#### 2.2.3. Procedure

**Figure 2.**
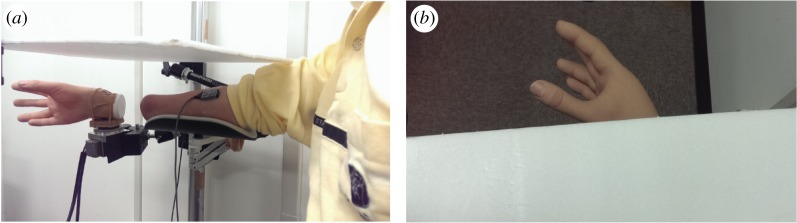
Experimental set-up for amputee participants. (*a*) The robotic arm was placed in front of the amputee participant's stump. EMG signals were recorded from the participant's wrist flexors/extensors to control the robotic arm. (*b*) Participant's-eye view of the experimental set-up with the participant watching the robotic arm.

The trans-radial amputee sat in front of the robotic arm (figure 2*a*). The participant's amputee stump was placed near the robotic arm in a natural posture. A plastic board was placed over the amputee's stump in order to cover the space between the robotic arm and the participant (figure 2*b*). The participants took part in the in-phase/out-of-phase movement experimental conditions for 5 min each. Considering the amputee's muscle fatigue, the experimental time was reduced relative to that of the able-bodied participants. In the conditions, each participant was instructed to contract their wrist flexors/extensors alternately, and to watch the robotic arm moving. In the in-phase movement condition, the wrist of the robotic arm was flexed/extended when the participant's flexor/extensor muscles for wrist movement were contracted. In the out-of-phase movement condition, the robotic arm moved in the opposite direction, that is, the wrist of the robotic arm flexed/extended when the participant's extensor/flexor muscles for wrist movement contracted. Each participant took part in the experiment six times.

#### 2.2.4. Psychological evaluation

Measurements of embodiment were performed immediately after each experiment. The SO and SA were evaluated with the same subjective ratings (−3 to +3) [15] as used for the able-bodied participants. Proprioceptive drift was not measured.

#### 2.2.5. Data analyses

Our *a priori*-defined criterion for a participant experiencing illusory ownership or agency was that a median score in the six experiments on the ownership or agency ratings was significantly greater than 0. We used the Wilcoxon signed-rank test at the 5% significance level. For comparisons between the in-phase and out-of-phase movement conditions, a Friedman test was used at the 5% significance level. For comparisons between the in-phase and out-of-phase movement conditions in each amputee participant, we used a Wilcoxon rank sum test at the 5% significance level.

## 3. Results

### 3.1. Able-bodied participants

**Figure 3.**
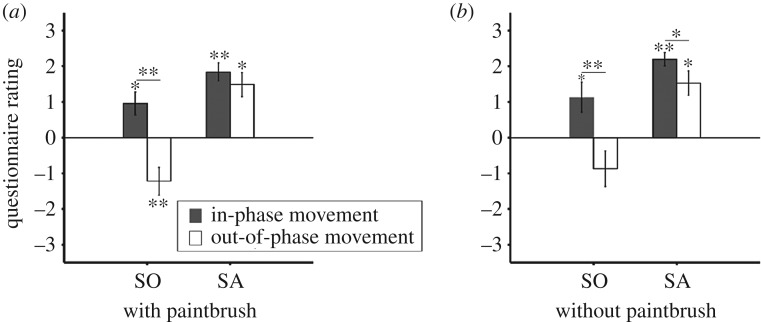
Psychological evaluation of ownership and agency for able-bodied participants. (*a*) Mean group scores on the ownership and agency ratings for the in-phase and out-of-phase movement conditions with the paintbrush. The able-bodied participants experienced illusory ownership and agency in the in-phase movement paintbrush condition. (*b*) Mean group scores on the ownership and agency ratings for the in-phase and out-of-phase movement conditions without the paintbrush. The able-bodied participants also experienced illusory ownership and agency in the in-phase movement condition without the paintbrush. Error bars show standard errors. Asterisks indicate statistically significant differences (*p < 0.05, **p < 0.01).

We prepared an in-house electromyographically controlled robotic arm in an RHI task. Able-bodied participants experienced illusory ownership and agency in the in-phase movement conditions with or without the paintbrush. The median group score on the ownership rating was significantly greater than 0 in both the in-phase movement condition with the paintbrush (median = +1.3, *V* = 91, *n* = 15, *p* = 0.014, Wilcoxon signed-rank test) and the in-phase movement condition without it (median = +1.3, *V* = 100, *n* = 15, *p* = 0.020; figure 3). There was no significant difference between the ownership score with versus without the paintbrush in the in-phase movement conditions. The median group score on the agency rating was significantly greater than 0 in the in-phase movement conditions both with (median = +2.0, *V* = 119, *n* = 15, *p* < 0.001) and without the paintbrush (median = +2.3, *V* = 120, *n* = 15, *p* < 0.001). The median group scores on the ownership ratings were significantly greater than their control ratings [15] in the in-phase movement condition (*V* = 105, *n* = 15, *p* < 0.001 with paintbrush; *V* = 105, *n* = 15, *p* < 0.001 without paintbrush). The median group scores on the agency ratings were significantly greater than their control ratings in the in-phase movement condition (*V* = 120, *n* = 15, *p* < 0.001 with paintbrushes; *V* = 103.5, *n* = 15, *p* < 0.001 without paintbrushes). In the out-of-phase movement conditions, subjective ratings of the SO decreased and were not significantly greater than 0, and were even significantly lower than 0 with the paintbrush (median = −1.7, *V* = 16, *n* = 15, *p* = 0.0099). By contrast, subjective ratings of the SA were greater than 0 both with (median = +2.0, *V* = 112, *n* = 15, *p* = 0.0015) and without the paintbrush (median = +1.3, *V* = 114, *n* = 15, *p* < 0.001). Subjective ratings of ownership or agency in the in-phase movement condition were compared to those ratings obtained in the out-of-phase movement condition. The median group score on the ownership ratings in the in-phase movement condition was significantly greater than reported in the out-of-phase movement condition with the paintbrush (*V* = 102, *n* = 15, *p* < 0.001) and the out-of-phase movement condition without the paintbrush (*V* = 89, *n* = 15, *p* < 0.001). The median group score on the agency ratings in the in-phase movement condition was also significantly greater than that reported in the out-of-phase movement condition without the paintbrush (*V* = 68, *n* = 15, *p* = 0.023), but not in the out-of-phase movement condition with the paintbrush (*V* = 33.5, *n* = 15, *p* = 0.21).

**Figure 4.**
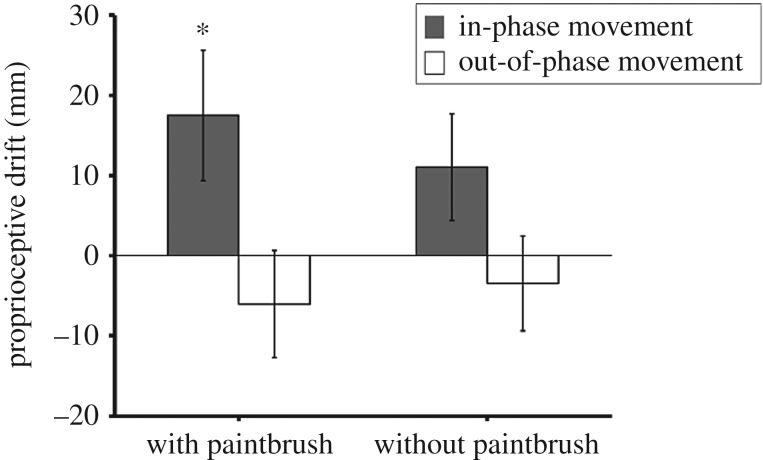
Proprioceptive drift data. A significant positive drift towards the robotic arm was observed in the in-phase movement condition with the paintbrush. Error bars show standard errors. Asterisk indicates statistically significant difference (*p < 0.05).

Proprioceptive drift was also assessed, and a significant positive proprioceptive drift was observed in the in-phase movement condition with the paintbrush (*V* = 96, *n* = 15, *p* = 0.040; figure 4). Note that the positive values indicated an upward drift in sensed hand position, towards the robotic arm.

### 3.2. Amputees

**Figure 5.**
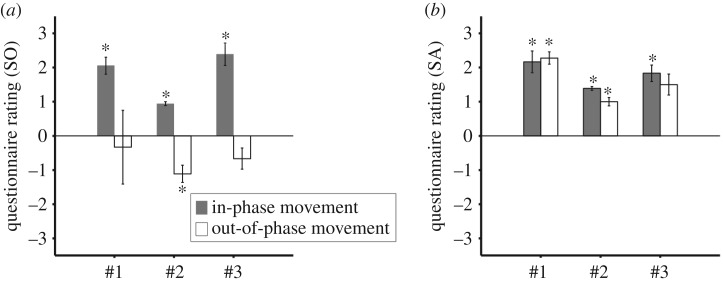
Psychological evaluation of ownership and agency for amputees. (*a*) The median scores on the agency ratings in the amputees were significantly greater than 0 in the in-phase movement condition. In the out-of-phase movement condition, ownership ratings were significantly lower than in the in-phase movement condition. (*b*) The median scores on the agency ratings in the amputees were significantly greater than 0 in the in-phase movement condition. In the out-of-phase movement condition, by contrast, the agency ratings were not significantly different (i.e. smaller) than in the in-phase movement condition. Error bars show standard errors. Asterisks indicate statistically significant differences (*p < 0.05).

The electromyographically controlled robotic arm was also applied to the amputees in an RHI task. The median score on the ownership rating in each amputee participant was significantly greater than 0 in the in-phase movement condition (*V* = 21, *n* = 6, *p* = 0.031 in participants #1 (median = +2.0), #2 (median = +1.0) and #3 (median = +2.7), Wilcoxon signed-rank test; figure 5*a*). The median score on the agency rating in each amputee was significantly greater than 0 in the in-phase movement condition (*V* = 21, *n* = 6, *p* = 0.031 in participants #1 (median = +2.5), #2 (median = +1.3) and #3 (median = +1.7); figure 5*b*). The results from the questionnaire revealed that the three amputees experienced illusory ownership and agency. The median group score on the ownership ratings were significantly greater than their control ratings [15] in the in-phase movement condition in each amputee (*V* = 21, *n* = 6, *p* = 0.031 in participants #1, #2 and #3). The median group score on the agency ratings were significantly greater than their control ratings in the in-phase movement condition in each amputee (*V* = 21, *n* = 6, *p* = 0.031 in participants #1, #2 and #3). The participant (#1) reporting phantom sensation did not experience any obvious change in the sensation following the experience of using the robotic arm.

The ratings of ownership in the in-phase movement condition were significantly higher than in the out-of-phase movement condition ($\chi_{\left(2\right)}^{2}=15.66,$ *p* < 0.001, Friedman test). We compared the ownership ratings between in-phase and out-of-phase movement conditions in each amputee. The ratings of ownership in the in-phase movement condition were significantly larger than those in the out-of-phase movement condition in #2 (*W* = 57, *n* = 6, *p* = 0.0066, Bonferroni corrected, Wilcoxon rank sum test) and #3 (*W* = 57, *n* = 6, *p* = 0.0066). By contrast, the ratings of agency in the in-phase movement condition were not significantly greater than those in the out-of-phase movement condition (*χ*^2^ (2) = 2.59, *p* = 0.11), and the median score on the agency rating was significantly greater than 0 in participants #1 and #2 (*V* = 21, *n* = 6, *p* = 0.031 in participants #1 (median = +2.3) and #2 (median = +1.0); figure 5*b*).

## 4. Discussion

In the present study, we prepared a robotic arm using proportional myoelectric control whereby the user could vary the joint positions continuously in an RHI task. EMG signals were recorded from each participant's arm, and the robotic arm, placed in front of the participant, was controlled. Subjective ratings of the SO and SA increased significantly, not only in the able-bodied participants but also in the three amputees.

These results reveal for the first time that the SO and the SA were extended to an electromyographically (EMG)-controlled robotic arm not only in able-bodied participants but also in amputees. In previous studies on the moving RHI, able-bodied participants were recruited, and the rubber hand was moved by a wooden rod connected to the participant's body [11–13,15,16] or by robotic technology in the form of a master–slave telemanipulator [14,28]. Note that such experimental conditions cannot be applied to amputees.

It is worth noting that some studies on the RHI in amputees have already been published. Ehrsson *et al*. [19], for instance, used synchronous touches to the stump and to the index finger of a rubber hand, and reported changes in SO. Meanwhile, Marasco *et al*. [21] indicated that amputees experienced SO over a prosthetic limb with physiologically appropriate cutaneous feedback from the prosthetic limb. Additionally, Rosen *et al*. [22] showed that amputees experienced a SO over an artificial hand by applying synchronous touches to their stump and the artificial hand, although they did not show that the amputees experienced a SA over the artificial hand. The results of the present study demonstrated that not only was the SO extended to an EMG-controlled robotic arm in the amputees but also the SA.

Using a detached EMG-controlled robotic hand in able-bodied participants, Romano *et al*. [18] reported that a shift in proprioceptive localization of the participant's own hand towards the robotic hand was induced under those conditions in which the real and robot hands opened and closed synchronously. However, no modulation of the SO or SA was observed under synchronous or asynchronous conditions. The former study and the present one applied somewhat different control paradigms to EMG-controlled robotic hands. The SO and SA were evaluated over a robotic arm using proportional myoelectric control [25], whereby the user could vary the joint positions continuously. In contrast, Romano *et al*. evaluated the SO and SA over a robotic arm using on–off myoelectric control, whereby the user could change discrete joint positions. Thus, it would seem likely that the user's fingers were in a different position from the robotic fingers in some locations. Moreover, the robotic arm in this study moved smoothly, after appropriate calibration, whereas the robotic arm in the previous study made a jerky movement because the output of the classifier was unstable [18]. This may also be caused by the difference in degrees of freedom. Thus, control methods for operating prosthetics may be important for the successful alteration of the SO/SA in amputees.

In the original RHI, watching a rubber hand being stroked while one's own unseen hand is stroked synchronously elicits the feeling that the rubber hand is one's own. Asynchronous stimulation of the two hands typically eliminates or reduces the illusion [4]. In the present study and recent studies on the moving RHI, SO and SA were extended to a rubber hand when able-bodied participants moved a rubber hand synchronously with their own body [11–13,15,16]. Out-of-phase movement of the rubber hand decreases SO over the rubber hand. Thus, the spatial congruency between the visual and somatosensory information is important when it comes to eliciting the illusion of ownership. The participants experienced a SA over the rubber hand even during the out-of-phase movement of the rubber hand, perhaps because the visual and somatosensory information was synchronous in time and the visual feedback of the wrist movements was predictable. These may be consistent with formerly proposed models of body ownership and agency, in that spatial consistency with the internal models of the body is related to eliciting the SO [7,13,29], and that the comparison between predicted and actual sensory feedback is related to the SA [30].

Our study also investigates the effect of tactile stimulation using a paintbrush, because a former study by Rosen *et al*. [22] reported that additional tactile stimulation with a brush affected the illusion of ownership, but our results showed no significant difference between the ownership score with versus without the paintbrush in the in-phase movement condition. This suggests that the additional tactile information was not critical for the illusion of ownership in our experimental setting.

The incorporation of non-body objects has been investigated in the fields of neuroscience and psychology [23,31]. In animal studies, Shokur *et al*. [32] recorded cortical neuronal activities in monkeys observing an avatar arm being touched by a virtual ball. They reported that, following a period when virtual touches occurred synchronously with physical brushes of the avatar arms, neurons in primary somatosensory cortex (S1) and motor cortex (M1) started to respond to the virtual touches without real touch [32]. Recently, our group used rubber tails in order to investigate the SO in rodents. We demonstrated that when the real tails and rubber tails were stroked synchronously, the mice would respond as if their own tails had been touched when the rubber tails were grasped; in contrast, when the stimuli were delivered asynchronously, the mean response rate was significantly lower when the rubber tail was grasped [33].

The incorporation of tools into the body representation has also been investigated. In animal research, Iriki *et al*. [35] trained a macaque monkey to retrieve distant objects using a rake and recorded neuronal activity in the caudal postcentral gyrus where somatosensory and visual signals converge. They reported that, during tool use, the visual receptive fields could be altered to include the entire length of the rake or at least to cover the expanded accessible region of space. The results of monkey electrophysiology, therefore, suggest that the phenomenon found here is related to tool manipulation.

In humans, when people cross their arms over their body midline, the subjective rank ordering of successive unseen tactile stimuli delivered to both hands can be affected and is often reversed at small interstimulus intervals [36–38]. Yamamoto & Kitazawa [39] applied a tactile temporal order judgement task to able-bodied participants and showed that the somatosensory signals evoked at the hands referred to the spatial locations of the tips of the drumsticks after holding them. The human psychophysical study suggested that the tool incorporation could be investigated using the tactile temporal order judgement task. In our recent study, we applied the task to amputees and demonstrated that they felt the tactile stimuli as having originated from the tip of the prosthetic arm [40], which suggested that the incorporation of the prosthetic arm could be investigated using the task.

The results of the present study showed for the first time that SO and SA were extended to an EMG-controlled robotic arm in amputees, suggesting that it is possible for amputees to alter the boundary of their body image to the tip of the prosthetic arm. One question that remains concerns whether the nature of the incorporation might be different between able-bodied participants and amputees; for able-bodied participants who already have an arm, incorporation may imply transfer from one's own body to the other; meanwhile, for amputees who have lost an arm, incorporation may imply the reproduction of a formerly-owned body part. Another point that may be interesting to consider here is that phantom limb sensation may interfere with the RHI in amputees, as suggested in a previous study [19]. Further studies in this area may contribute to understanding the mechanism that causes SO/SA and may help to realize a myoelectric prosthesis that is not only a tool but an integrated body part for amputees.

## Data accessibility

Data are available from the Dryad Digital Repository: http://dx.doi.org/10.5061/dryad.6675p [41].
